# Immune evasion: An imperative and consequence of MYC deregulation

**DOI:** 10.1002/1878-0261.13695

**Published:** 2024-07-02

**Authors:** Bastian Krenz, Jongkuen Lee, Toshitha Kannan, Martin Eilers

**Affiliations:** ^1^ Department of Biochemistry and Molecular Biology Theodor Boveri Institute, Biocenter, University of Würzburg Würzburg Germany; ^2^ Mildred Scheel Early Career Center Würzburg Germany; ^3^ Comprehensive Cancer Center Mainfranken Würzburg Germany

**Keywords:** immune checkpoints, immune evasion, immunotherapy, MYC, MYC inhibition, stress resilience, tumor metabolism

## Abstract

MYC has been implicated in the pathogenesis of a wide range of human tumors and has been described for many years as a transcription factor that regulates genes with pleiotropic functions to promote tumorigenic growth. However, despite extensive efforts to identify specific target genes of MYC that alone could be responsible for promoting tumorigenesis, the field is yet to reach a consensus whether this is the crucial function of MYC. Recent work shifts the view on MYC's function from being a gene‐specific transcription factor to an essential stress resilience factor. In highly proliferating cells, MYC preserves cell integrity by promoting DNA repair at core promoters, protecting stalled replication forks, and/or preventing transcription‐replication conflicts. Furthermore, an increasing body of evidence demonstrates that MYC not only promotes tumorigenesis by driving cell‐autonomous growth, but also enables tumors to evade the host's immune system. In this review, we summarize our current understanding of how MYC impairs antitumor immunity and why this function is evolutionarily hard‐wired to the biology of the MYC protein family. We show why the cell‐autonomous and immune evasive functions of MYC are mutually dependent and discuss ways to target MYC proteins in cancer therapy.

## Graphical abstract

The MYC protein family is deregulated in most human tumors and regulates the transcription of genes to promote tumorigenic growth in immunocompetent hosts. In this review, we integrate current knowledge on the cell‐autonomous functions of MYC as a nuclear protein and its critical ability to promote immune escape, explaining why deregulated transcription and immune escape go hand in hand.

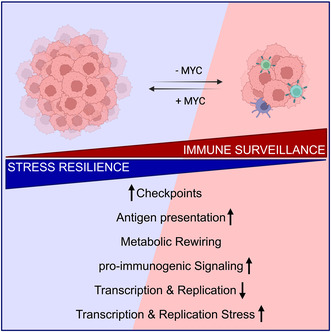

**Abbreviations**
bHLHZ: basic helix–loop–helix‐zipper
CAF: cancer associated fibroblasts
DAMPs: damage‐associated molecular patterns
GLS: glutaminase
GLUT1: glucose transporter 1
HK2: hexokinase 2
IDR: intrinsically disordered regions
LDHA: lactate dehydrogenase A
MAPK: mitogen‐activated protein kinase
MCT1: monocarboxylate transporter 1
NAD+: nicotinamide adenine dinucleotide
ORF: open reading frame
PDAC: pancreatic ductal adenocarcinoma
PRR: pattern recognition receptors
TCA: tricarboxylic acid
TME: tumor microenvironment
TNBC: triple‐negative breast cancer

## 1 MYC proteins: A ubiquitous family in cancer

The MYC family is comprised of three paralogs, MYC, MYCN, and MYCL [1]. The overall structure and domain organization of MYC family members are conserved and numerous studies support the idea that these three proteins are, to a certain extent, functionally redundant. MYC expression is commonly upregulated in proliferating or dividing cells and plays a crucial role in the embryonic development and pluripotency [2, 3].

**Fig. 1:**
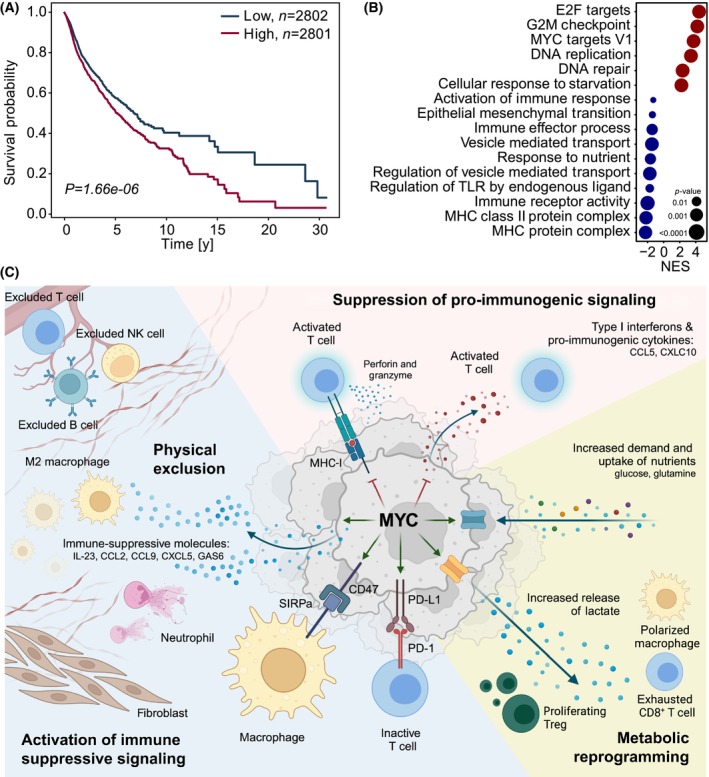
MYC is a major driver of immune evasion. (A) High expression of MYC is associated with worse overall survival in a panel of human cancer patients (*N* = 5603). (B) High expression of MYC correlates with upregulation of MYC‐target genes, and gene sets associated with growth and proliferation as well as starvation. Low expression of MYC is associated with activation of immunity, vesicular transport, and antigen presentation (*N* = 5603). (C) MYC promotes immune evasion in tumors via different mechanisms, ranging from activation or inhibition of specific genes to metabolic reprogramming of the tumor microenvironment.

While deregulated MYC expression is pervasive in human cancer and is associated with poor prognosis (Fig. 1A), expression of MYCN is more restricted to neural lineages and MYCL is expressed in the lung and mature dendritic cells, implying their specific roles in different tissues and development stages [4, 5, 6, 7, 8]. MYC protein levels in tumor cells are increased by genetic alterations, including translocation as described in Burkitt's lymphoma and multiple myeloma [9, 10] or amplifications in multiple cancer types [11]. Additionally, MYC is strongly upregulated by oncogenic activation of one or more signaling pathways, including the Wnt/β‐catenin, Notch, and mitogen‐activated protein kinase (MAPK) pathways [12, 13].

Most of MYC's functions are attributed to its distinctive ability to bind DNA as a heterodimer together with its partner‐protein MAX. By doing so, MYC proteins promote pause release and activate the transcription of genes [14, 15, 16]. Even though MYC does not dimerize with basic helix–loop–helix‐zipper (bHLHZ) proteins other than MAX, MAX can form heterodimers with other bHLHZ proteins, such as the MXD family and MGA to repress transcription [17]. Furthermore, a MAX‐like bHLHZ protein known as MLX participates in dimerization with the MXD family, MondoA and ChREBP, to modulate a wide range of transcriptional programs in response to extracellular and intracellular signals, including mitogenic and metabolic signals [17]. Additionally, MYC interacts at supraphysiological levels with the zinc finger containing protein MIZ1, forming a repressive complex that limits the expression of certain genes, some of which are also implicated to be involved in immune surveillance [16, 18, 19, 20, 21, 22, 23]. By binding to MAX or MIZ1 at oncogenic levels, MYC has been shown to integrate multiple pro‐tumorigenic cellular signals and orchestrate a broad range of biological activities and processes, such as cell growth, proliferation, and energy metabolism [12] (Fig. 1B). Chromatin binding assays in cells with deregulated expression of MYC show that MYC proteins invade close to all promoters of genes transcribed by RNAPII. Yet, RNA sequencing analyses show that the activation of MYC only causes mild changes in the global gene expression profile of a cell, implying that gene regulation is not necessarily directly correlated with DNA‐binding of MYC and might not be the only relevant function of MYC proteins in a cell [1].

Mass spectrometry analyses of MYC immunoprecipitations or proximity‐dependent labelling assays identify proteins of versatile function that bind to MYC or are at least in close proximity to MYC in the cell. Besides its well‐described association with factors that are involved in transcriptional initiation, there is growing evidence that MYC proteins interact with factors that are involved in chromatin architecture and remodeling, DNA damage repair, transcriptional termination, and RNA biology [24, 25, 26, 27, 28].

Functional studies analyzing these interactors have revealed a complex role of MYC proteins in increasing the stress resilience of a cell by reducing torsional stress at core promoters [29, 30], limiting R loop formation [14], coordinating transcription and replication [24, 31, 32], and RNA processing [33, 34, 35]. Recently, different laboratories have shown that MYC, a bona fide DNA‐binding protein, also binds RNA molecules, fundamentally changing our understanding of MYC molecules [35, 36, 37]. However, the large protein interactome of MYC and the functional interactions among the network members that are crucial for MYC's biological functions are well documented elsewhere [1, 38, 39, 40].

Yet, most reviews discussing the role of MYC proteins in immune evasion limit themselves to focus on the function of MYC as a regulator of the transcription of specific genes that impact the interaction of tumor cells with the immune system, ignoring that MYC proteins have a large and complex interactome, allowing MYC to be involved in immune evasive functions that are independent of the transcriptional regulation of specific genes. The objective of this review is to integrate the state‐of‐the‐art knowledge on both of MYC's roles in cancer: its ability to increase the immune privilege of tumors in the cellular ecosystem and its intracellular function as a regulator of transcription and a stress resilience factor.

## 2 Immune evasion in cancer

Cancer is characterized by autonomous outgrowth driven by genetic and epigenetic alterations. In many cases, mutations in signaling cascades cause constitutive activation of growth‐promoting pathways, making cell proliferation and cell growth independent of growth factors and external stimuli. Additional mutations in tumor suppressors accelerate deregulated growth by increasing DNA damage events in the cell which lead to the accumulation of further mutations. These diverse alterations in the genome may eventually give rise to an autologous and invasive growth of tumor cells, but present also serious obstacles that activate innate immunity and the integrated stress response. This circumstance can restrict or prevent the tumor outgrowth in an immunocompetent individual: For instance, the multitude of genetic alterations in tumors often leads to the expression of mutated proteins that are presented as digested peptides via the major histocompatibility complexes MHC class I and II on the surface of the cell [41, 42]. These neoantigens can be recognized by cells of the adaptive immune system, especially T cells, leading to the elimination of the tumor cells [43]. Furthermore, the deregulated growth of tumors is associated with an increased frequency of DNA replication, a shorter S phase and a higher transcriptional rate throughout all phases of the cell cycle [44, 45, 46]. Transcription and DNA replication are inherently stressful processes that occur on the same template. Increased rates of both processes and can therefore lead to an abnormal accumulation of different nucleic acid byproducts, which challenge the cell by activating intracellular pattern recognition receptors, leading to pro‐immunogenic signaling and ultimately the expression of immunologic messenger molecules [47, 48, 49].

Together, neoantigen presentation and the release of interferons, cytokines, and chemokines attract diverse immune cells that have the potential to eradicate tumor cells and consequently limit tumorigenic growth. To enable deregulated outgrowth, it is crucial for tumors to overcome this bottleneck. This can clearly been shown in animal models lacking immune cells and, as a consequence, are more prone to cancer formation and progression if challenged with carcinogenic substances compared with immunocompetent models [50].

Various mechanisms have been described to explain how cancer cells obviate recognition and elimination by the immune system. These include downregulation of neoantigen presentation by genetic or epigenetic alterations, for example, via the polycomb repressive complex 2 (PRC2) or by decreasing the stability of the MHC class I complex on the cell surface [51, 52], transcriptional upregulation of immune checkpoints [53], increased metabolism of DAMPs [21, 54, 55], direct interference with intracellular pro‐immunogenic signaling [56], or co‐deletion of loci encoding for type I interferons [57]. MYC proteins have been described to be involved in all these processes integrating two major features of tumor cells: autonomous, the deregulated growth of the cells, and evasion of tumor cells from immune privilege in the tumor ecosystem.

## 3 How do MYC proteins cool down the tumor ecosystem?

In the tumor ecosystem, cancer cells constantly interact with numerous neighboring cells, causing significant changes in their behaviors. During cancer initiation and development, this reciprocal and dynamic communication between cancer cells and surrounding cells plays a crucial role in fostering cancer cell survival, angiogenesis, invasion, and metastatic dissemination [58]. This intercellular crosstalk is complex and works not only via physical cell–cell contacts but also via paracrine signaling through the mutual release of multiple cytokines, chemokines, and growth factors, leading to the remodeling and evolution of the TME [59]. The role of MYC proteins in the regulation of these intercellular interactions is of particular interest: Numerous studies have shown that targeting MYC in mouse models is more efficient and has a more persistent effect in immunocompetent mice compared with immune‐deficient mice [21, 60, 61]. Additionally, the deregulation of MYC proteins has been shown to be associated with cold tumor ecosystems and causes exclusion or dysfunction of cytotoxic lymphocytes in the TME of human patients [62, 63, 64, 65].

Very early during carcinogenesis, tumor cells are eliminated by the immune system due to their pro‐immunogenic signaling or presentation of neoantigens (elimination phase). Later, the depletion of tumor suppressors, the activation of oncogenes, and the increasing ability of tumor cells to evade immune surveillance cause transformed cells to be not efficiently eradicated by immune‐mediated killing anymore (equilibrium phase). Finally, tumor cells gain the fitness that allows them to bypass the immune pressure and facilitates their uncontrolled outgrowth in the escape phase [66]. This concept is called ‘cancer immunoediting’ and postulates that the selection pressure exerted by the immune system shapes the immunogenicity of cancers by promoting engraftment of clones with strong immune evasive potential [66]. MYC contributes to the immune escape of tumor cells by fostering cell‐autonomous growth and by immunoediting the tumor cells to allow for immune privilege in the tumor ecosystem.

Currently, the field agrees that interfering with MYC function strongly effects the tumor's ability to evade the immune system. However, the immunological responses that have been observed after targeting MYC either genetically or pharmacologically seem to be very different. MYC depletion in a KPC mouse model (*Kras* ^*G12D/+*^; *p53* ^*R172H/+*^) for pancreatic ductal adenocarcinoma (PDAC) activates T cells [21, 32], whereas in other mouse models, B and NK cells have been shown to mediate regression of tumors [22, 67, 68]. In other studies, deregulated expression of MYC in tumor cells has been shown to influence the polarization of macrophages [69]. Given the fact that the activity of different immune cells is controlled by diverse signaling molecules, the above observations can only be explained if deregulated MYC controls the expression of distinct genes in different tumor types and in tumors with different mutational backgrounds.

### 3.1 MYC proteins restrict neoantigen presentation

Major histocompatibility class I (MHC‐I) molecules are heterodimers composed of two polypeptide chains, α and β_2_‐microglobulin [70]. MHC‐I molecules are present on the surface of virtually all nucleated cells and display peptides derived from intracellular proteins to effector T cells. This process is essential for reporting pathophysiological alterations in a cell to the immune system since they indicate a pathogen invasion or an oncogenic insult. Neoantigen presentation enables the immune system to identify cells that harbor ‘foreign proteins’ and allows for specific eradication of these cells [71]. In most cases, tumor cells display neoantigens that originate from somatic mutations or RNA‐based variants, making them potential targets of T cells [41, 72]. Thus, it is not surprising that multiple tumor types tend to impair antigen processing and presentation to escape from the immune recognition and elimination. Indeed, downregulation of MHC‐I on the cell surface has been reported in a wide range of cancers and was shown to often correlate with a decreased number of tumor infiltrating lymphocytes and a poor prognosis for patients [73].

Suppression of neoantigen presentation was historically the first described immune evasive function of MYC (Fig. 1C). Bernards et al. and Versteeg et al. [74, 75] both reported an inverse correlation between expression of MYC family proteins and MHC‐I expression levels. Overexpression of MYC in human melanoma and of MYCN in rat neuroblastoma was shown to downregulate the levels of MHC‐I and promote tumor growth *in vivo* [74]. Acute reduction in MYC protein levels in lymphoblastoid cells induces a strong MHC class I‐dependent immunogenicity of the cells [76]. Similarly, depletion of MYC using short hairpin RNAs causes transcriptional upregulation of murine MHC class I genes and increases antigen presentation in a murine pancreatic cancer model [21]. Depleting MYC in triple‐negative breast cancer (TNBC) cells increases antigen presentation via transcriptional upregulation of the whole antigen presenting machinery [77]. In line with this, elevated MYC expression in B‐cell lymphoma also dampens the MHC‐II pathway, which is crucial for the presentation of antigens to CD4+ T cells and initiation of immune responses [78]. More specifically, MYC‐overexpressing B‐cell lymphoma displays with defects in the expression of MHC class II antigen processing and presenting machinery (APM) components. Conversely, MYC inhibition enhanced the expression of the HLA‐DM (human MHC class II) protein and partially restored functional antigen presentation of tumor cells to CD4+ T cells, emphasizing the strong regulatory impact of MYC on the antigen recognition pathway [78].

Taking advantage of these results, first pharmacological approaches were successfully tested to restore neoantigen presentation on the cell surface, thereby sensitizing tumors to T cell‐dependent killing. Examples of tested compounds are Palbociclib, EZH2 inhibitors, or Birinapant (mimetic of second mitochondria‐derived activator of caspase) [51, 79, 80, 81]. This shows that restoring neoantigen presentation is a therapeutic opportunity and that repression of MHC‐I and in some cases also MHC‐II in tumors is indeed crucial for immunogenic escape.

### 3.2 MYC proteins regulate expression of immune checkpoints and chemokines

In lymphoma cells, MYC activates the expression of PD‐L1 (programmed death ligand 1), a key inhibitory signal that represents an adaptive immune tolerance mechanism, by directly binding to the core promoter of the gene [60] (Fig. 1C). PD‐L1 is present on the surface of these tumor cells and can interact with PD‐1 (programmed cell death protein 1) on T cells. This interaction leads to T‐cell anergy, a hyporesponsive state in which lymphocytes are functionally inactive to antigens or even induce T cell apoptosis [82]. Depletion of MYC in lymphomas causes decrease in PD‐L1 levels and results in a regression of the tumors in immunocompetent mice. Importantly, restoring PD‐L1 expression in a MYC‐depleted background in this model promotes tumor development, showing that in these tumors MYC is responsible for immune evasion via upregulation of PD‐L1 [60]. Accordingly, MYC and PD‐L1 expressions were found to significantly correlate in different tumor entities and that MYC transcriptionally controls PD‐L1 expression [83, 84]. This correlation was further emphasized by the observation that translation of the PD‐L1 transcript is inhibited in *KRAS* ^*G12D*^‐induced liver tumors by non‐canonical upstream open reading frame (ORF) in its 5′‐untranslated region (UTR), which is circumvented by transgenic overexpression of MYC in these *KRAS* ^*G12D*^ tumors [85].

Additionally, cancer cells express specific immune checkpoints, to prevent the elimination by innate immune cells. These immune checkpoints are regulatory proteins of the immune system. One of the best‐characterized innate immune checkpoints is the CD47‐signal regulatory protein α (SIRPα) axis, commonly regarded as a ‘don't eat me’ signal [86]. SIRPα is expressed on the surface of myeloid cells, such as macrophages, monocytes, and myeloid dendritic cells [87, 88]. Similar to PD‐L1, MYC binds to the promoter of the CD47 gene in lymphoma cells to activate its transcription [60] (Fig. 1C). However, the MYC‐dependent regulation of immune checkpoints seems to be dependent on the tumor type and the mutational background. While MYC binds to the core promoter of PD‐L1 and/or CD47 and regulates their transcription in some models [60, 83, 84, 89], neither chromatin occupancy of MYC in these loci nor regulation of the genes are present in murine PDAC cells from the KPC mouse model [16, 21].

Numerous studies have revealed that oncogenic MYC influences the expression of chemokines and cytokines, modulating the composition of the ecosystem and the functionality of different immune cell types [90]. For example, MYC inactivation in TNBC result in the upregulation of innate immune cytokines, such as CCL5, CXCL10, and IFN‐β [91]. MYC cooperates with TWIST1 to induce the secretions of CCL2 and IL‐13 that recruit and polarize tumor associated macrophages, resulting in enhanced metastasis in a model of hepatocellular carcinoma [92]. Likewise, in a mouse model of *KRAS* ^*G12D*^‐driven lung adenoma, MYC has been described to promote IL‐23 and CCL9 expression to recruit macrophages, promote angiogenesis, and exclude cytotoxic lymphocytes from the tumor [89] (Fig. 1C). In a mouse model of pancreatic cancer, the correlation between CCL9 and MYC expression was confirmed; however, the mechanistic details described in this model were different, pointing out that immune suppression and composition of the TME is not solely dependent on MYC but is also dramatically influenced by the tumor origin and subtype. Additionally, MYC promotes GAS6 release in PDAC, a signaling molecule that is implicated to be immunosuppressive, and MYC fosters desmoplasia of pancreatic tissue [67, 93] (Fig. 1C). Desmoplasia is described to confer aggressiveness and to limit immune surveillance. While the complex role of cancer associated fibroblasts (CAF) is not the scope of this review, it is important to keep in mind that the extracellular matrix that is established by CAFs provides a physical barrier and limits infiltration with immune cells [94, 95, 96].

### 3.3 Mechanisms of MYC‐mediated transcriptional repression

Depending on the tumor type and the mouse model, different laboratories have described different target genes to be either positively or negatively regulated by MYC. While the transcriptional activation of genes is mediated by the MYC/MAX heterodimer, MYC can also restrict transcription by forming repressive complexes together with MIZ1 or it can be absent from the core promoter of these genes all together. Several studies imply that the cooperation of MYC and MIZ1 is crucial to suppress pro‐immunogenic signaling in tumors and promote tumor growth in an immunocompetent host [21, 22, 23, 69].

MYC and MIZ cooperatively bind to the core promoter of shared genes to restrict their transcription. Both the dysfunctional truncation of MIZ1 lacking the POZ domain (MIZ^ΔPOZ^) and the MYC protein carrying a point mutation (MYC^V394D^) that disrupts the interaction with MIZ1 activate genes that are jointly repressed by the MYC/MIZ heterodimer [18, 19, 97]. Early transcription of cell cycle checkpoint genes and TGF‐β signaling have been described to be confined by MYC and MIZ1 [98, 99]. However, global expression analyses of cells expressing MIZ^ΔPOZ^ and cells expressing MYC^V394D^ show that many more genes are restrained by MYC and MIZ1 [16, 19, 21]. A growing number of studies indicate that the repressive MYC/MIZ1 complex is crucial for the strong immune evasive capacity of MYC: In MYC‐driven pancreatic cancer, the interaction between MYC and MIZ1 attenuates the expression of interferons, limiting macrophage‐derived CXCL13 production and preventing infiltration of the tumor with NK and B cells [22]. Similarly, in TNBC, MYC and MIZ both bind to promoters to repress the expression of CCL5 and CXCL10, both crucial signaling molecules for immune cell chemotaxis [23]. Correspondingly, in hepatocellular carcinoma, chromatin occupancy of MYC and MIZ1 correlates with an attenuated expression of MHC class I genes [69]. Additionally, in an orthotopic transplant model of pancreatic cancer, MYC and MIZ1 attenuate the expression of genes involved in vesicular transport. This restrains the release of dsRNA containing extracellular vesicles and the recognition of immunogenic dsRNA by TLR3. Consequently, MYC/MIZ indirectly prevent the activation of the innate immunity in the tumor cell and downstream the expression of pro‐immunogenic genes [21] (Fig. 1C).

Despite varying target genes and mechanisms, the field concurs on the important role of the repressive MYC and MIZ1 heterodimer, proven by several genetic mouse models. In the pancreatic cancer mouse model system KPC (*Kras* ^*G12D/+*^; T*p53* ^*R172H/+*^; *Pdx1‐Cre*), MYC is haplo‐insufficient for the tumorigenesis, meaning that already the deletion of one allele of MYC restricted tumorigenesis and extended the survival of tumor bearing mice. Similarly, survival improved with the deletion of the POZ domain of MIZ1 in the same KPC mouse model, but also in a mouse model driven by MYC amplification and Kras^G12D^ activation [16, 22, 93].

Nonetheless, growing evidence suggests that there are other mechanisms of MYC‐dependent gene repression that contribute to immune evasion. While pause release is observed on all genes transcribed by RNA polymerase II after activation of MYC or MYCN [14, 15], the traveling ratio and transcription elongation is significantly different for MYC activated genes and MYC‐repressed genes, thereby shaping the gene expression of MYC‐deregulated tumor cells [14, 27, 32]. At oncogenic levels, MYC sequesters crucial elongation factors like SPT5 or SPT6 to MYC activated genes, and limits the amount of available SPT5 and SPT6 molecules [1, 27, 32, 100, 101].

In an orthotopic transplant model for pancreatic cancer, MYC together with its co‐factor CTR9 constrain the transcription of rather short, pro‐immunogenic genes by restricting elongation factors to long, MYC activated genes, for example, DNA damage repair genes. Depletion of CTR9 in mouse PDAC tumors results in a redistribution of the elongation factor SPT6 from long to short genes, thereby reducing the expression of DNA damage repair genes and promoting the transcription of shorter genes, including MHC class I proteins. Restoring neoantigen presentation also restores immune surveillance in mice PDAC tumors and facilitates T cell‐mediated killing of the tumor [32]. Comparably, activation of MYC in osteosarcoma cells leads to squelching of another elongation factor, SPT5, from genes with no or only moderate binding of MYC [1, 27]. Consequently, MYC limits the efficient expression of these genes by its absence from the core promoter. In different tumor types, specifically the core promoters of pro‐immunogenic genes appear to be deserts of MYC binding even at oncogenic MYC levels, detaining elongation factors from being recruited by MYC and thereby preventing a pro‐immunogenic gene signature [1, 21, 27].

### 3.4 Metabolic reprogramming by MYC: Cause or consequence?

The metabolic demands and the pathways involved in cellular activities are highly dynamic and rely heavily on the state of cells and the environmental cues, including growth factors and nutrient availability [102]. Unlike resting cells, rapidly dividing cells, particularly cancer cells, require abundant building blocks for macromolecular biosynthesis, generation of adenosine triphosphate (ATP), and maintenance of cellular redox status [103]. In this context, MYC enables cells to cope with these challenges by reprogramming the metabolic pathways and changing the production of intermediate metabolites. MYC induces the activation of various genes that support growth by regulating metabolic pathways such as glycolysis, glutaminolysis, ribosomal biogenesis, protein synthesis, and mitochondrial metabolism [104, 105, 106].

Metabolic stresses, like lack of nutrients or acidosis, imposed on immune cells within the TME, compromise their antitumor cytotoxicity, suggesting that deregulation of MYC in cancers can affect the host immune responses both directly and indirectly via metabolism [107, 108].

Numerous studies have reported that high MYC promotes an increased consumption of glucose, in part, by transcriptionally activating glucose transporter 1 (GLUT1), hexokinase 2 (HK2), and lactate dehydrogenase A (LDHA) [109, 110, 111] (Fig. 1C). LDHA is an enzyme that catalyzes pyruvate into lactate and is crucial for anaerobic glycolysis (‘the Warburg effect’) via regeneration of the electron acceptor nicotinamide adenine dinucleotide (NAD+) [112]. It is well documented that MYC overexpression in cancer cells enhances the levels of LDHA, leading to an increase in extracellular acidification [113, 114]. The acidic microenvironment, caused by lactate overproduction and secretion from malignant cells, influences the presence and function of different immune cell types, promoting the immunosuppressive TME [107]: Lactate exposure attenuates activation of dendritic cells, polarizes macrophages towards the M2‐like phenotype, limits cytolytic activity of NK cells, and suppresses T cell response [114, 115, 116, 117, 118]. Recently, regulatory T cells (Tregs) were shown to consume lactate via monocarboxylate transporter 1 (MCT1) and upregulate the expression of PD‐1 in MYC‐amplified tumors [119] (Fig. 1C).

In addition to glucose, high glutamine consumption rates in cancer cells have been repeatedly reported [120]. Glutamine is the most abundant circulating amino acid in human plasma and can be converted to glutamate and subsequently to α‐ketoglutarate (α‐KG), which is incorporated into the tricarboxylic acid (TCA) cycle for ATP production and the provision of intermediate metabolites for biosynthesis [105, 120]. MYC is actively engaged in glutaminolysis by upregulating the expression of glutaminase (GLS) and glutamine transporters, including SLC1A5 and SLC7A5/SLC3A2 [106, 121]. Glutamine metabolism has profound effects not only on tumor cells but also on immune cell activation, proliferation, and differentiation [122, 123, 124]. Therefore, glutamine deprivation in the tumor microenvironment (TME) induced by high uptake in cancer cells may affect the function and fate of immune cells [125]. For instance, it was shown that SLC1A5‐dependent glutamine uptake is required for induction of T helper 1 (Th1) cells, Th17 cells, and inflammatory T cell responses in mouse models [126]. Moreover, a lack of extracellular glutamine leads to a decrease in the intracellular amount of α‐KG, which skews the balance between the production of Th1 and Tregs towards the immunosuppressive Tregs [127]. Therefore, high energy and nutrient demand in growing cancer cells limits immune response.

There is also emerging evidence showing that functional crosstalk among other factors within the MYC network plays a significant role in alleviating cellular stress [38, 39]. MNT promotes MYC‐driven B cell lymphomagenesis by attenuating the level of pro‐apoptotic BIM [128]. In response to metabolic acidosis, MondoA, a nutrient‐sensing transcription factor, provides a negative feedback loop that maintains cellular energy homeostasis [129]. MondoA/MLX has also been shown to cope with metabolic stresses through lipid biosynthesis and suppress the death signaling pathways in MYC‐dependent tumors and spermatogenesis [130, 131], implying that an imbalance in the MYC network might affect the immune responses.

### 3.5 What goes around comes around: MYC fosters stress resilience

MYC promotes two inherently stressful processes, namely, transcription and replication, fostering cell cycle progression, and shortening the duration of the S phase [24, 31, 44, 132, 133]. Both processes are a potential source of molecular patterns (specifically aberrant nucleic acids) with a strong capacity to activate integrated stress response and the innate immunity. DNA fragments from stalling replication forks activate the cGAS‐STING axis [47, 49], DNA–RNA hybrid (R loop) formation can be a strong activator of sensors for aberrant RNA and DNA [134, 135], and the induction of transcription‐replication conflicts promotes upregulation of cGAS in murine neuroblastoma [31]. Additionally, dysregulation and aberrations in the transcriptional cycle and in RNA processing are a source of accumulating immunogenic RNA molecules. Limited or exhausted splicing and defective RNA decay increases dsRNA content in the cell [32, 48, 54, 55, 136]. The observation that highly immunogenic patterns accumulate in deregulated proliferating tumor cells and are an Achilles heel in tumor cells has led to the development of potential therapeutic approaches. Epigenetic therapies like inhibition of EZH2, DNMT, or HDAC, or deletion of ADAR1, a dsRNA‐editing enzyme, activate innate immunity and synergizes with checkpoint inhibition [137, 138, 139].

**Fig. 2:**
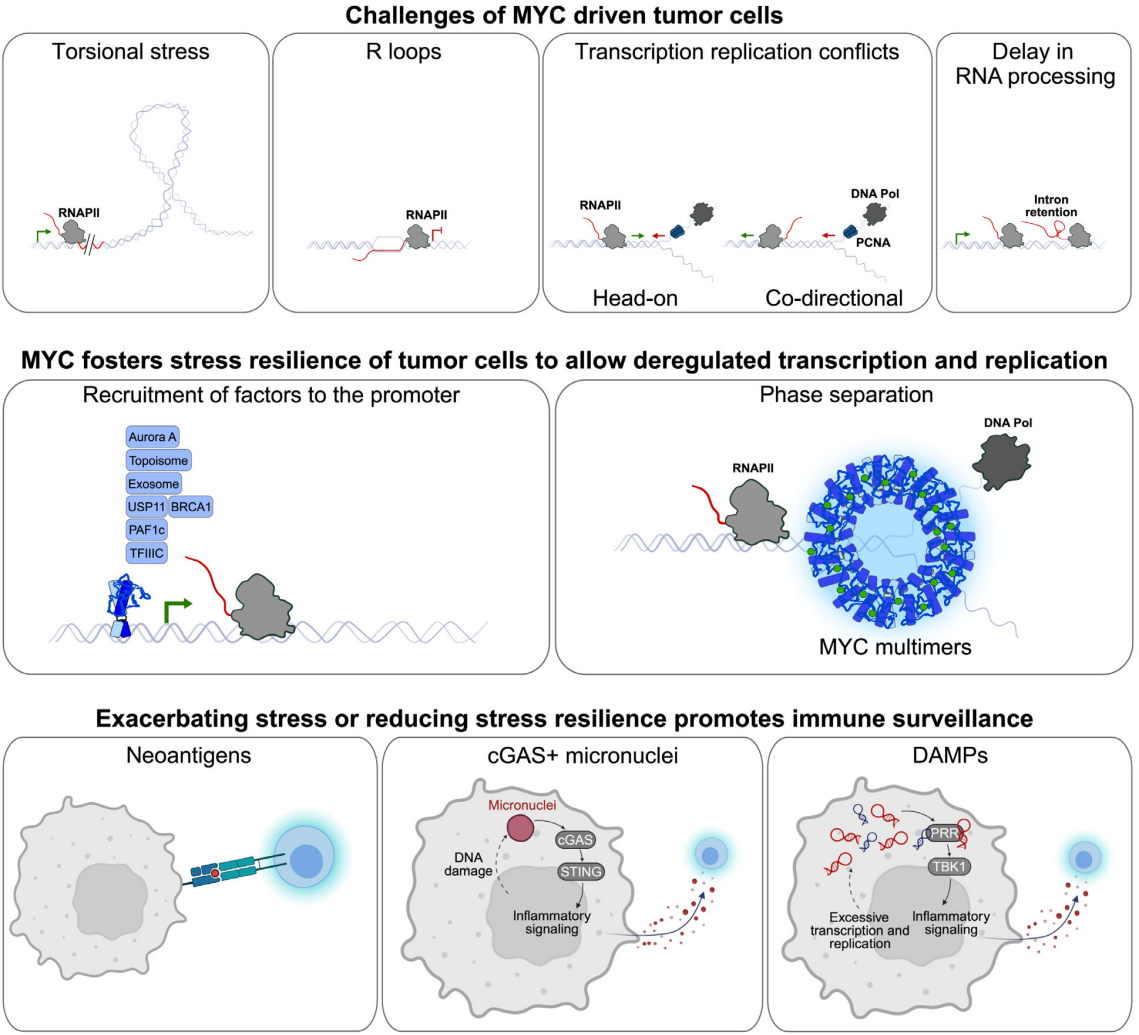
MYC as a master regulator and mediator of transcription and replication—two inherently stressful processes that utilize the same template. Deregulated transcription evokes challenges like torsional stress, R loops, transcription, and replication conflicts, as well as exhaustion of splicing capacity. MYC protects the cellular integrity by shaping and coordinating transcription and replication with its ability to bind and recruit manifold proteins and by phase separating around stalled replication forks. Targeting MYC results in increased cellular stress, being a source of mutations and neoantigens as well as DAMP‐driven immunity fostered by aberrant nucleic acids and micronuclei.

Due to their potential to recruit manifold protein complexes or to phase separate, MYC proteins increase resilience of highly proliferating cells to stress resulting from transcription and replication in different cell lines and tumor models [14, 24, 29, 30, 34, 35, 140] (Fig. 2): (a) Inefficient coupling of transcription and splicing can result in repetitive element‐derived dsRNA that engages PRRs in MYC‐driven TNBC and PDAC [21, 48, 136]. In this context, MYC was described to induce a pan‐cancer network of oncogenic splicing factors that promote splicing is crucial for lymphomagenesis [141, 142] and MYC‐driven tumor cells are highly sensitive to inhibition or interference with splicing [33, 48, 143, 144]. Inhibition of splicing or exhaustion of splicing capacity in MYC‐driven tumor cells induces accumulation of intron‐derived dsRNA that activates pattern recognition receptors [21, 48] or promotes presentation of neoantigens [145]. (b) DNA damage and its response are a source of fragmented DNA and DNA–RNA hybrids as well as micronuclei that can be recognized by AIM2, the cGAS‐STING axis or the corresponding TLRs [49, 134, 135, 146, 147, 148]. MYC proteins either recruit co‐factors to the promoter to foster RNA processing, DNA repair mechanisms, and to reduce torsional stress [29, 30, 34], or they phase separate to protect stalled replication forks [24]. MYCN recruits decapping complexes and the nuclear exosome to degrade nascent RNA and allows for transcriptional termination when facing the replication fork [14, 34]. TFIIIC, an architectural protein, controls and limits the presence of MYCN in hubs of active promoters to favor termination of non‐phosphorylated RNA polymerase II, taming the function of MYCN as activator of transcription [28]. Coordinating transcription and replication, stabilizing stalled replication forks, promoting fidelity of transcription, and fostering DNA damage repair increase stress resilience in tumor. Thereby, MYC potentially prevents the accumulation of mutations that are a potential source of neoantigens, as well as the accumulation of aberrant nucleic acids with a strong potential to activate the immune system.

## 4 Unleashing antitumor immunity by targeting MYC

MYC has been regarded as a ‘undruggable’ transcription factor that contains large intrinsically disordered regions (IDR) and lacks a binding pocket or specific enzymatic activity, complicating or even impeding structure‐guided drug design approaches [149]. However, there have been a lot of attempts to modulate MYC directly and indirectly. Originally MYC proteins were targeted due to their strong potential in driving proliferation of human cells in culture, but genetic depletion of MYC in mouse models made it clear, that a strong and persistent effect of MYC depletion *in vivo* is dependent on the immune system [21, 32, 61]. Pleiotropic interactors of MYC proteins have been described in the past years, and inhibiting these surrogate targets, which are critical mediators of context dependent MYC functions, has shown antitumor effects also *in vivo*. Combined inhibition of Aurora kinase A (AURKA), a kinase that interacts with MYCN during S phase, and ATR (ataxia telangiectasia and Rad3 related), a protein activated by DNA damage events, causes immune‐dependent regression of neuroblastoma in a highly aggressive mouse model [26, 31]. MYC hyperactivation upregulates precursor messenger RNA (pre‐mRNA) synthetic activities and overloads the core spliceosome that is required to process pre‐mRNA [141, 150]. This leaves MYC‐driven tumors vulnerable to inhibition of the co‐transcriptional RNA processing and splicing by targeting the likes of PRMT5 or BUD31 [142, 151]. Intriguingly, spliceosome‐targeted therapies in MYC‐driven breast cancer trigger antiviral immune responses [48].

Small molecules that interfere with the interface of MYC and MAX or MYC and MIZ1 to either downregulate MYC‐driven transcription or upregulate MYC‐repressed genes are also currently under investigation. Interfering with the dimerization of MYC and MAX using small molecules shows immune‐dependent antitumor effects [152, 153, 154, 155]. Targeting the repressive MYC/MIZ1‐complex or the POZ domain of MIZ1 might allow for immune‐mediated killing of tumors [156, 157]. Depleting MYC with BET/BRD4 (Bromodomain and extra‐terminal domain) inhibitors that displace BRD4 from the super‐enhancers around the MYC locus induce cell cycle arrest and antitumor immunity [158]. The Omomyc mini‐protein, a dominant negative allele of MYC with promising results in cell culture and mouse models, is now in Phase I trials [159, 160]. By competing with MYC and MAX at the core promoter, Omomyc homodimers limit oncogenic transcription by MYC [161]. Treatment of mice with the Omomyc peptide OMO‐103 in a lung cancer mouse model as well as samples from OMO‐103‐treated patients showed engagement of the immune system [162].

Targeting MYC in mouse models activates the immune system towards the tumor, but survival is in most cases only extended without achieving full remission. However, MYC inhibition synergizes with immunotherapeutic approaches and extends survival. Immune checkpoint blockade combined with JQ‐1, a BET/BRD4 inhibitor, induces recurrence‐free survival in a substantial fraction of mice with osteosarcoma [65]. Targeting the MYC/MAX interaction in an immune‐competent transplant model for prostate cancer or melanoma retards tumor growth in combination with immune checkpoint blockade [152, 155].

## 5 Conclusions and perspectives

The ability of MYC to regulate the transcription cycle and its involvement in fundamental cell‐intrinsic biological processes, including cellular growth, proliferation, and metabolism, has been regarded as the prevailing means by which it drives cancer progression. Intracellularly, MYC coordinates and stimulates transcription and replication, two important processes in the cell, that use the same template. Intercellularly, MYC allows the immune privilege of tumor cells in the ecosystem. These two functions of MYC proteins are discussed separately for most of the time, but there is an increasing number of indications and evidence, that these both functions are evolutionary tied together. We hypothesize that in MYC‐deregulated tumors with increased rates of transcription, replication, and very short cell cycle, tumors face several obstacles (e.g., transcription‐replication‐conflicts, torsional stress, neoantigen presentation, DNA damage at active promoters) that make immune evasion an imperative for tumor cells. By modulating intracellular pathways and shaping the transcription, MYC meets its responsibility to hide tumor cells from the immune system. Analysis on immune cell profiling in human melanoma and neuroblastoma showed that MYC or MYCN amplification, respectively, was strongly correlated with the suppressed immune phenotypes and impaired tumor immunity [158, 163].

**Fig. 3:**
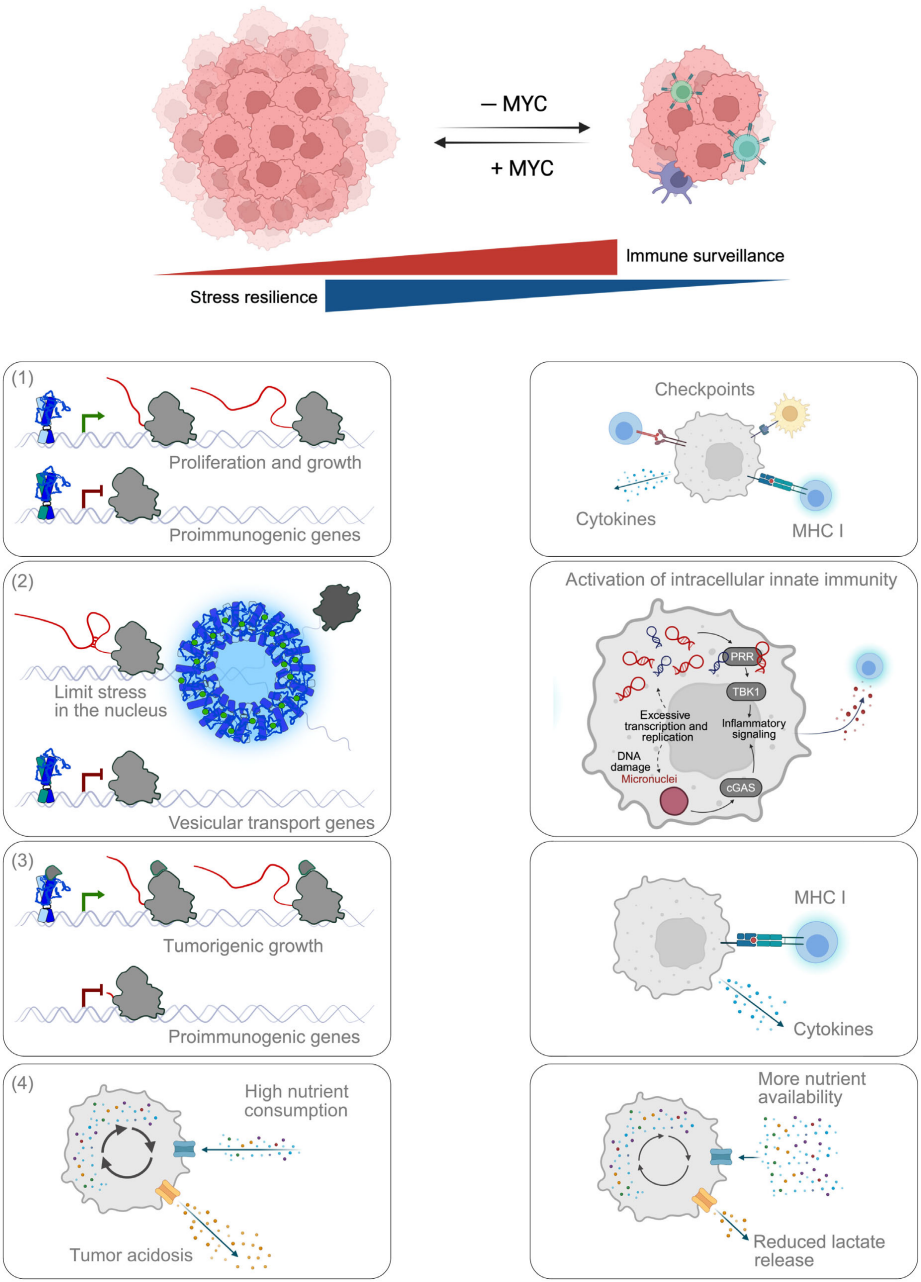
MYC proteins increase stress resilience in tumor cells and reduce immune visibility. Several different mechanisms of MYC‐driven immune evasion have been described: (1) By binding to the core promoter of genes and the recruitment of MAX and MIZ1, MYC proteins activate and repress transcription of genes that regulate the immune visibility of tumor cells, for example, neoantigen presentation, cytokines, and interferons, as well as immune checkpoints. (2) MYC proteins recruit factors to the core promoter or phase separate thereby restricting stress in the tumor cells leading to DNA damage and stress signaling. Additionally, repression of the metabolism of aberrant nucleic acids by the MYC/MIZ1 heterodimer limits immune visibility. (3) By sequestering crucial elongation factors to pro‐tumorigenic genes and squelching factors from pro‐immunogenic genes, MYC suppresses NF‐κB signaling and antigen presentation. (4) MYC‐mediated growth of tumors is accompanied by metabolic reprogramming and increased consumption of nutrients by tumor cells, thereby depriving immune cells of essential nutrients in the tumor microenvironment. In parallel, MYC promotes the release of immune suppressive lactate.

Despite the widely accepted view that MYC is a key regulator of immune evasion, a process that is crucial for tumorigenic growth *in vivo*, the proposed concepts of how MYC facilitates this essential function are rather diverse (Fig. 3): (a) A number of publications showed that MYC proteins directly bind to the core promoter of immune‐related genes to regulate their transcription [22, 23]. (b) MYC proteins repress the activation of pro‐immunogenic signaling by preventing the activation of innate immunity by damage‐associated molecular patterns (DAMPs). Both accumulation and transport of DAMPs are limited by oncogenic levels of MYC [21, 26]. (c) While MYC is invading at oncogenic levels close to all promoters of genes that are actively transcribed by RNA polymerase II, some studies show that pro‐immunogenic genes are deserts for MYC binding and that the absence of MYC at the core promoter of specific genes restricts their transcription [1, 21, 27, 32, 101].

Still, none of the models solely explain how MYC proteins facilitate immune evasion throughout many different tumor types and across species. Looking for a consensus target gene in tumors of different types and with different mutational background is probably effortless, since the transcriptional programs that allow for deregulated growth widely vary [164]. However, there is an increasing number of pharmacological interventions in the preclinical state that show promising effects on targeting MYC's function as a driver of tumorigenic growth and immune evasion, striking an impression that 45 years after its discovery we are getting closer to target MYC‐driven tumors in patients.

## Conflict of interest

ME is a cofounder of Tucana.

## Author contributions

BK, JL, and ME conceived and wrote the manuscript following an invitation from Prof. Kevin Ryan. TK performed bioinformatic analysis of TCGA data.

## References

[1] Baluapuri A , Wolf E , Eilers M . Target gene‐independent functions of MYC oncoproteins. Nat Rev Mol Cell Biol. 2020;21(5):255–267. 32071436 10.1038/s41580-020-0215-2 PMC7611238

[2] Zimmerman KA , Yancopoulos GD , Collum RG , Smith RK , Kohl NE , Denis KA , et al. Differential expression of myc family genes during murine development. Nature. 1986;319(6056):780–783. 2419762 10.1038/319780a0

[3] Takahashi K , Yamanaka S . Induction of pluripotent stem cells from mouse embryonic and adult fibroblast cultures by defined factors. Cell. 2006;126(4):663–676. 16904174 10.1016/j.cell.2006.07.024

[4] Malynn BA , de Alboran IM , O'Hagan RC , Bronson R , Davidson L , DePinho RA , et al. N‐myc can functionally replace c‐myc in murine development, cellular growth, and differentiation. Genes Dev. 2000;14(11):1390–1399. 10837031 PMC316670

[5] Kohl NE , Kanda N , Schreck RR , Bruns G , Latt SA , Gilbert F , et al. Transposition and amplification of oncogene‐related sequences in human neuroblastomas. Cell. 1983;35(2 Pt 1):359–367. 6197179 10.1016/0092-8674(83)90169-1

[6] Schwab M , Alitalo K , Klempnauer KH , Varmus HE , Bishop JM , Gilbert F , et al. Amplified DNA with limited homology to myc cellular oncogene is shared by human neuroblastoma cell lines and a neuroblastoma tumour. Nature. 1983;305(5931):245–248. 6888561 10.1038/305245a0

[7] Nau MM , Brooks BJ , Battey J , Sausville E , Gazdar AF , Kirsch IR , et al. L‐myc, a new myc‐related gene amplified and expressed in human small cell lung cancer. Nature. 1985;318(6041):69–73. 2997622 10.1038/318069a0

[8] Anderson DA , Ou F , Kim S , Murphy TL , Murphy KM . Transition from cMyc to L‐Myc during dendritic cell development coordinated by rising levels of IRF8. J Exp Med. 2022;219(2):e20211483. 34958351 10.1084/jem.20211483 PMC8713298

[9] Mikulasova A , Ashby CC , Tytarenko RG , Bauer M , Mavrommatis K , Wardell CP , et al. MYC rearrangements in multiple myeloma are complex, can involve more than five different chromosomes, and correlate with increased Expression of MYC and a distinct downstream gene Expression pattern. Blood. 2017;130(Suppl 1):65.

[10] Schmitz R , Ceribelli M , Pittaluga S , Wright G , Staudt LM . Oncogenic mechanisms in Burkitt lymphoma. Cold Spring Harb Perspect Med. 2014;4(2):a014282. 24492847 10.1101/cshperspect.a014282 PMC3904095

[11] Meyer N , Penn LZ . Reflecting on 25 years with MYC. Nat Rev Cancer. 2008;8(12):976–990. 19029958 10.1038/nrc2231

[12] Dang CV . MYC on the path to cancer. Cell. 2012;149(1):22–35. 22464321 10.1016/j.cell.2012.03.003 PMC3345192

[13] Vaseva AV , Blake DR , Gilbert TSK , Ng S , Hostetter G , Azam SH , et al. KRAS suppression‐induced degradation of MYC is antagonized by a MEK5‐ERK5 compensatory mechanism. Cancer Cell. 2018;34(5):807–822. e7. 30423298 10.1016/j.ccell.2018.10.001 PMC6321749

[14] Herold S , Kalb J , Büchel G , Ade CP , Baluapuri A , Xu J , et al. Recruitment of BRCA1 limits MYCN‐driven accumulation of stalled RNA polymerase. Nature. 2019;567(7749):545–549. 30894746 10.1038/s41586-019-1030-9 PMC7611299

[15] Rahl PB , Lin CY , Seila AC , Flynn RA , McCuine S , Burge CB , et al. c‐Myc regulates transcriptional pause release. Cell. 2010;141(3):432–445. 20434984 10.1016/j.cell.2010.03.030 PMC2864022

[16] Walz S , Lorenzin F , Morton J , Wiese KE , von Eyss B , Herold S , et al. Activation and repression by oncogenic MYC shape tumour‐specific gene expression profiles. Nature. 2014;511(7510):483–487. 25043018 10.1038/nature13473 PMC6879323

[17] O'Shea JM , Ayer DE . Coordination of nutrient availability and utilization by MAX‐ and MLX‐centered transcription networks. Cold Spring Harb Perspect Med. 2013;3(9):a014258. 24003245 10.1101/cshperspect.a014258 PMC3753723

[18] Wiese KE , Haikala HM , von Eyss B , Wolf E , Esnault C , Rosenwald A , et al. Repression of SRF target genes is critical for Myc‐dependent apoptosis of epithelial cells. EMBO J. 2015;34(11):1554–1571. 25896507 10.15252/embj.201490467 PMC4474530

[19] Wiese KE , Walz S , von Eyss B , Wolf E , Athineos D , Sansom O , et al. The role of MIZ‐1 in MYC‐dependent tumorigenesis. Cold Spring Harb Perspect Med. 2013;3(12):a014290. 24296348 10.1101/cshperspect.a014290 PMC3839600

[20] Gebhardt A , Frye M , Herold S , Benitah SA , Braun K , Samans B , et al. Myc regulates keratinocyte adhesion and differentiation via complex formation with Miz1. J Cell Biol. 2006;172(1):139–149. 16391002 10.1083/jcb.200506057 PMC2063541

[21] Krenz B , Gebhardt‐Wolf A , Ade CP , Gaballa A , Roehrig F , Vendelova E , et al. MYC‐ and MIZ1‐dependent vesicular transport of double‐strand RNA controls immune evasion in pancreatic ductal adenocarcinoma. Cancer Res. 2021;81:4242–4256. 34145038 10.1158/0008-5472.CAN-21-1677 PMC7611539

[22] Muthalagu N , Monteverde T , Raffo‐Iraolagoitia X , Wiesheu R , Whyte D , Hedley A , et al. Repression of the type I interferon pathway underlies MYC‐ and KRAS‐dependent evasion of NK and B cells in pancreatic ductal adenocarcinoma. Cancer Discov. 2020;10(6):872–887. 32200350 10.1158/2159-8290.CD-19-0620 PMC7611248

[23] Zimmerli D , Brambillasca CS , Talens F , Bhin J , Linstra R , Romanens L , et al. MYC promotes immune‐suppression in triple‐negative breast cancer via inhibition of interferon signaling. Nat Commun. 2022;13(1):6579. 36323660 10.1038/s41467-022-34000-6 PMC9630413

[24] Solvie D , Baluapuri A , Uhl L , Fleischhauer D , Endres T , Papadopoulos D , et al. MYC multimers shield stalled replication forks from RNA polymerase. Nature. 2022;612(7938):148–155. 36424410 10.1038/s41586-022-05469-4

[25] Kalkat M , Resetca D , Lourenco C , Chan PK , Wei Y , Shiah YJ , et al. MYC protein Interactome profiling reveals functionally distinct regions that cooperate to drive tumorigenesis. Mol Cell. 2018;72(5):836–848.e7. 30415952 10.1016/j.molcel.2018.09.031

[26] Büchel G , Carstensen A , Mak KY , Roeschert I , Leen E , Sumara O , et al. Association with Aurora‐a controls N‐MYC‐dependent promoter escape and pause release of RNA polymerase II during the cell cycle. Cell Rep. 2017;21(12):3483–3497. 29262328 10.1016/j.celrep.2017.11.090 PMC5746598

[27] Baluapuri A , Hofstetter J , Dudvarski Stankovic N , Endres T , Bhandare P , Vos SM , et al. MYC recruits SPT5 to RNA polymerase II to promote Processive transcription elongation. Mol Cell. 2019;74(4):674–687.e11. 30928206 10.1016/j.molcel.2019.02.031 PMC6527870

[28] Vidal R , Leen E , Herold S , Müller M , Fleischhauer D , Schülein‐Völk C , et al. Association with TFIIIC limits MYCN localization in hubs of active promoters and chromatin accumulation of non‐phosphorylated RNA polymerase II. Cold Spring Harbor Laboratory; 2023. 10.7554/eLife.94407 PMC11343564 39177021

[29] Endres T , Solvie D , Heidelberger JB , Andrioletti V , Baluapuri A , Ade CP , et al. Ubiquitylation of MYC couples transcription elongation with double‐strand break repair at active promoters. Mol Cell. 2021;81(4):830–844.e13. 33453168 10.1016/j.molcel.2020.12.035 PMC7611325

[30] Das SK , Kuzin V , Cameron DP , Sanford S , Jha RK , Nie Z , et al. MYC assembles and stimulates topoisomerases 1 and 2 in a “topoisome”. Mol Cell. 2022;82(1):140–158.e12. 34890565 10.1016/j.molcel.2021.11.016 PMC8750365

[31] Roeschert I , Poon E , Henssen AG , Dorado Garcia H , Gatti M , Giansanti C , et al. Combined inhibition of Aurora‐a and ATR kinases results in regression of MYCN‐amplified neuroblastoma. Nat Can. 2021;2(3):312–326. 10.1038/s43018-020-00171-8 PMC7610389 33768209

[32] Gaballa A , Gebhardt‐Wolf A , Krenz B , Mattavelli G , John M , Cossa G , et al. PAF1c links S‐phase progression to immune evasion and MYC function in pancreatic carcinoma. Nat Commun. 2024;15(1):1446. 38365788 10.1038/s41467-024-45760-8 PMC10873513

[33] Cossa G , Roeschert I , Prinz F , Baluapuri A , Silveira Vidal R , Schülein‐Völk C , et al. Localized inhibition of protein phosphatase 1 by NUAK1 promotes spliceosome activity and reveals a MYC‐sensitive feedback control of transcription. Mol Cell. 2020;77(6):1322–1339.e11. 32006464 10.1016/j.molcel.2020.01.008 PMC7086158

[34] Papadopoulos D , Solvie D , Baluapuri A , Endres T , Ha SA , Herold S , et al. MYCN recruits the nuclear exosome complex to RNA polymerase II to prevent transcription‐replication conflicts. Mol Cell. 2022;82(1):159–176.e12. 34847357 10.1016/j.molcel.2021.11.002

[35] Papadopoulos D , Ha SA , Fleischhauer D , Uhl L , Russell TJ , Mikicic I , et al. The MYCN oncoprotein is an RNA‐binding accessory factor of the nuclear exosome targeting complex. Mol Cell. 2024;84:2070–2086.e20. 38703770 10.1016/j.molcel.2024.04.007

[36] Oksuz O , Henninger JE , Warneford‐Thomson R , Zheng MM , Erb H , Vancura A , et al. Transcription factors interact with RNA to regulate genes. Mol Cell. 2023;83(14):2449–2463.e13. 37402367 10.1016/j.molcel.2023.06.012 PMC10529847

[37] Yang L , Pratihar S , Horste EH , Mitschka S , Mey ASJS , Al‐Hashimi HM , et al. mRNA interactions with disordered regions control protein activity. bioRxiv. 2023. 10.1101/2023.02.18.529068

[38] Conacci‐Sorrell M , McFerrin L , Eisenman RN . An overview of MYC and its interactome. Cold Spring Harb Perspect Med. 2014;4(1):a014357. 24384812 10.1101/cshperspect.a014357 PMC3869278

[39] Carroll PA , Freie BW , Mathsyaraja H , Eisenman RN . The MYC transcription factor network: balancing metabolism, proliferation and oncogenesis. Front Med. 2018;12(4):412–425. 30054853 10.1007/s11684-018-0650-z PMC7358075

[40] Papadopoulos D , Uhl L , Ha SA , Eilers M . Beyond gene expression: how MYC relieves transcription stress. Trends Cancer. 2023;9(10):805–816. 37422352 10.1016/j.trecan.2023.06.008

[41] Tretter C , de Andrade Krätzig N , Pecoraro M , Lange S , Seifert P , von Frankenberg C , et al. Proteogenomic analysis reveals RNA as a source for tumor‐agnostic neoantigen identification. Nat Commun. 2023;14(1):4632. 37532709 10.1038/s41467-023-39570-7 PMC10397250

[42] Xie N , Shen G , Gao W , Huang Z , Huang C , Fu L . Neoantigens: promising targets for cancer therapy. Signal Transduct Target Ther. 2023;8(1):9. 36604431 10.1038/s41392-022-01270-x PMC9816309

[43] Bergmann C , Lowenstein P . MHC class I expression and CD8 T cell function: Towards the cell biology of T‐APC interactions in the infected brain. In: Lane TE , Carson M , Bergmann C , Wyss‐Coray T , editors. Central Nervous System Diseases and Inflammation. Boston, MA: Springer US; 2008. p. 277–306.

[44] Macheret M , Halazonetis TD . Intragenic origins due to short G1 phases underlie oncogene‐induced DNA replication stress. Nature. 2018;555(7694):112–116. 29466339 10.1038/nature25507 PMC5837010

[45] Bradner JE , Hnisz D , Young RA . Transcriptional addiction in cancer. Cell. 2017;168(4):629–643. 28187285 10.1016/j.cell.2016.12.013 PMC5308559

[46] da Costa AABA , Chowdhury D , Shapiro GI , D'Andrea AD , Konstantinopoulos PA . Targeting replication stress in cancer therapy. Nat Rev Drug Discov. 2023;22(1):38–58. 36202931 10.1038/s41573-022-00558-5 PMC11132912

[47] Coquel F , Silva MJ , Técher H , Zadorozhny K , Sharma S , Nieminuszczy J , et al. SAMHD1 acts at stalled replication forks to prevent interferon induction. Nature. 2018;557(7703):57–61. 29670289 10.1038/s41586-018-0050-1

[48] Bowling EA , Wang JH , Gong F , Wu W , Neill NJ , Kim IS , et al. Spliceosome‐targeted therapies trigger an antiviral immune response in triple‐negative breast cancer. Cell. 2021;184(2):384–403.e21. 33450205 10.1016/j.cell.2020.12.031 PMC8635244

[49] Emam A , Wu X , Xu S , Wang L , Liu S , Wang B . Stalled replication fork protection limits cGAS–STING and P‐body‐dependent innate immune signalling. Nat Cell Biol. 2022;24(7):1154–1164. 35817959 10.1038/s41556-022-00950-8 PMC9924303

[50] Shankaran V , Ikeda H , Bruce AT , White JM , Swanson PE , Old LJ , et al. IFNgamma and lymphocytes prevent primary tumour development and shape tumour immunogenicity. Nature. 2001;410(6832):1107–1111. 11323675 10.1038/35074122

[51] Burr ML , Sparbier CE , Chan KL , Chan YC , Kersbergen A , Lam EYN , et al. An evolutionarily conserved function of Polycomb silences the MHC Class I antigen presentation pathway and enables immune evasion in cancer. Cancer Cell. 2019;36(4):385–401.e8. 31564637 10.1016/j.ccell.2019.08.008 PMC6876280

[52] Yamamoto K , Venida A , Yano J , Biancur DE , Kakiuchi M , Gupta S , et al. Autophagy promotes immune evasion of pancreatic cancer by degrading MHC‐I. Nature. 2020;581(7806):100–105. 32376951 10.1038/s41586-020-2229-5 PMC7296553

[53] Boussiotis VA . Molecular and biochemical aspects of the PD‐1 checkpoint pathway. N Engl J Med. 2016;375(18):1767–1778. 27806234 10.1056/NEJMra1514296 PMC5575761

[54] Ghosh S , Guimaraes JC , Lanzafame M , Schmidt A , Syed AP , Dimitriades B , et al. Prevention of dsRNA‐induced interferon signaling by AGO1x is linked to breast cancer cell proliferation. EMBO J. 2020;39(18):e103922. 32812257 10.15252/embj.2019103922 PMC7507497

[55] Wu Y , Zhao W , Liu Y , Tan X , Li X , Zou Q , et al. Function of HNRNPC in breast cancer cells by controlling the dsRNA‐induced interferon response. EMBO J. 2018;37(23):e99017. 30158112 10.15252/embj.201899017 PMC6276880

[56] Ghosh M , Saha S , Bettke J , Nagar R , Parrales A , Iwakuma T , et al. Mutant p53 suppresses innate immune signaling to promote tumorigenesis. Cancer Cell. 2021;39(4):494–508.e5. 33545063 10.1016/j.ccell.2021.01.003 PMC8044023

[57] Barriga FM , Tsanov KM , Ho YJ , Sohail N , Zhang A , Baslan T , et al. MACHETE identifies interferon‐encompassing chromosome 9p21.3 deletions as mediators of immune evasion and metastasis. Nature Cancer. 2022;3:1367–1385. 36344707 10.1038/s43018-022-00443-5 PMC9701143

[58] Jin MZ , Jin WL . The updated landscape of tumor microenvironment and drug repurposing. Signal Transduct Target Ther. 2020;5(1):166. 32843638 10.1038/s41392-020-00280-x PMC7447642

[59] de Visser KE , Joyce JA . The evolving tumor microenvironment from cancer initiation to metastatic outgrowth. Cancer Cell. 2023;41(3):374–403. 36917948 10.1016/j.ccell.2023.02.016

[60] Casey SC , Tong L , Li Y , do R , Walz S , Fitzgerald KN , et al. MYC regulates the antitumor immune response through CD47 and PD‐L1. Science. 2016;352(6282):227–231. 26966191 10.1126/science.aac9935 PMC4940030

[61] Rakhra K , Bachireddy P , Zabuawala T , Zeiser R , Xu L , Kopelman A , et al. CD4(+) T cells contribute to the remodeling of the microenvironment required for sustained tumor regression upon oncogene inactivation. Cancer Cell. 2010;18(5):485–498. 21035406 10.1016/j.ccr.2010.10.002 PMC2991103

[62] Layer JP , Kronmüller MT , Quast T , Boorn‐Konijnenberg D , Effern M , Hinze D , et al. Amplification of N‐Myc is associated with a T‐cell‐poor microenvironment in metastatic neuroblastoma restraining interferon pathway activity and chemokine expression. Onco Targets Ther. 2017;6(6):e1320626. 10.1080/2162402X.2017.1320626 PMC5486176 28680756

[63] Wolpaw AJ , Grossmann LD , Dessau JL , Dong MM , Aaron BJ , Brafford PA , et al. Epigenetic state determines inflammatory sensing in neuroblastoma. Proc Natl Acad Sci. 2022;119(6):e2102358119. 35121657 10.1073/pnas.2102358119 PMC8832972

[64] Kacher J , Manches O , Aspord C , Sartelet H , Chaperot L . Impaired antitumor immune response in MYCN‐amplified Neuroblastoma is associated with lack of CCL2 secretion and poor dendritic cell recruitment. Cancer Research Communications. 2022;2(7):577–589. 36923280 10.1158/2767-9764.CRC-21-0134 PMC10010397

[65] Jiang K , Zhang Q , Fan Y , Li J , Zhang J , Wang W , et al. MYC inhibition reprograms tumor immune microenvironment by recruiting T lymphocytes and activating the CD40/CD40L system in osteosarcoma. Cell Death Dis. 2022;8(1):117. 10.1038/s41420-022-00923-8 PMC8924240 35292660

[66] Schreiber RD , Old LJ , Smyth MJ . Cancer immunoediting: integrating immunity's roles in cancer suppression and promotion. Science. 2011;331(6024):1565–1570. 21436444 10.1126/science.1203486

[67] Sodir NM , Kortlever RM , Barthet VJA , Campos T , Pellegrinet L , Kupczak S , et al. MYC instructs and maintains pancreatic adenocarcinoma phenotype. Cancer Discov. 2020;10:588–607. 31941709 10.1158/2159-8290.CD-19-0435

[68] Swaminathan S , Hansen AS , Heftdal LD , Dhanasekaran R , Deutzmann A , Fernandez WDM , et al. MYC functions as a switch for natural killer cell‐mediated immune surveillance of lymphoid malignancies. Nat Commun. 2020;11(1):2860. 32503978 10.1038/s41467-020-16447-7 PMC7275060

[69] Dhanasekaran R , Hansen AS , Park J , Lemaitre L , Lai I , Adeniji N , et al. MYC overexpression drives immune evasion in hepatocellular carcinoma that is reversible through restoration of Proinflammatory macrophages. Cancer Res. 2023;83(4):626–640. 36525476 10.1158/0008-5472.CAN-22-0232 PMC9931653

[70] Dhatchinamoorthy K , Colbert JD , Rock KL . Cancer immune evasion through loss of MHC Class I antigen presentation. Front Immunol. 2021;12:636568. 33767702 10.3389/fimmu.2021.636568 PMC7986854

[71] Wieczorek M , Abualrous ET , Sticht J , Álvaro‐Benito M , Stolzenberg S , Noé F , et al. Major histocompatibility complex (MHC) Class I and MHC Class II proteins: conformational plasticity in antigen presentation. Front Immunol. 2017;8:292. 28367149 10.3389/fimmu.2017.00292 PMC5355494

[72] Gupta RG , Li F , Roszik J , Lizée G . Exploiting tumor Neoantigens to target cancer evolution: current challenges and promising therapeutic approaches. Cancer Discov. 2021;11(5):1024–1039. 33722796 10.1158/2159-8290.CD-20-1575 PMC8102318

[73] Cornel AM , Mimpen IL , Nierkens S . MHC Class I downregulation in cancer: underlying mechanisms and potential targets for cancer immunotherapy. Cancer. 2020;12(7):1760. 10.3390/cancers12071760 PMC7409324 32630675

[74] Bernards R , Dessain SK , Weinberg RA . N‐Myc amplification causes down‐modulation of MHC class‐I antigen expression in Neuroblastoma. Cell. 1986;47(5):667–674. 3096575 10.1016/0092-8674(86)90509-x

[75] Versteeg R , Noordermeer IA , Krüse‐Wolters M , Ruiter DJ , Schrier PI . c‐myc down‐regulates class HLA expression in human melanomas. EMBO J. 1988;7:1023–1029. 3402430 10.1002/j.1460-2075.1988.tb02909.x PMC454430

[76] Staege MS , Lee SP , Frisan T , Mautner J , Scholz S , Pajic A , et al. MYC overexpression imposes a nonimmunogenic phenotype on Epstein‐Barr virus‐infected B cells. Proc Natl Acad Sci USA. 2002;99(7):4550–4555. 11917131 10.1073/pnas.072495599 PMC123685

[77] Lee JV , Housley F , Yau C , Nakagawa R , Winkler J , Anttila JM , et al. Combinatorial immunotherapies overcome MYC‐driven immune evasion in triple negative breast cancer. Nat Commun. 2022;13(1):3671. 35760778 10.1038/s41467-022-31238-y PMC9237085

[78] God JM , Cameron C , Figueroa J , Amria S , Hossain A , Kempkes B , et al. Elevation of c‐MYC disrupts HLA class II‐mediated immune recognition of human B cell tumors. J Immunol. 2015;194(4):1434–1445. 25595783 10.4049/jimmunol.1402382 PMC4323931

[79] Gu SS , Zhang W , Wang X , Jiang P , Traugh N , Li Z , et al. Therapeutically increasing MHC‐I Expression potentiates immune checkpoint blockade. Cancer Discov. 2021;11(6):1524–1541. 33589424 10.1158/2159-8290.CD-20-0812 PMC8543117

[80] Zhou L , Mudianto T , Ma X , Riley R , Uppaluri R . Targeting EZH2 enhances antigen presentation, antitumor immunity, and circumvents anti‐PD‐1 resistance in head and neck cancer. Clin Cancer Res. 2020;26(1):290–300. 31562203 10.1158/1078-0432.CCR-19-1351 PMC6942613

[81] Goel S , DeCristo MJ , Watt AC , BrinJones H , Sceneay J , Li BB , et al. CDK4/6 inhibition triggers anti‐tumour immunity. Nature. 2017;548(7668):471–475. 28813415 10.1038/nature23465 PMC5570667

[82] Chen L . Co‐inhibitory molecules of the B7‐CD28 family in the control of T‐cell immunity. Nat Rev Immunol. 2004;4(5):336–347. 15122199 10.1038/nri1349

[83] Kim EY , Kim A , Kim SK , Chang YS . MYC expression correlates with PD‐L1 expression in non‐small cell lung cancer. Lung Cancer. 2017;110:63–67. 28676221 10.1016/j.lungcan.2017.06.006

[84] Liang MQ , Yu FQ , Chen C . C‐Myc regulates PD‐L1 expression in esophageal squamous cell carcinoma. Am J Transl Res. 2020;12(2):379–388. 32194890 PMC7061834

[85] Xu YC , Poggio M , Jin HY , Shi Z , Forester CM , Wang Y , et al. Translation control of the immune checkpoint in cancer and its therapeutic targeting. Nat Med. 2019;25(2):301. 30643286 10.1038/s41591-018-0321-2 PMC6613562

[86] Feng M , Jiang W , Kim BYS , Zhang CC , Fu YX , Weissman IL . Phagocytosis checkpoints as new targets for cancer immunotherapy. Nat Rev Cancer. 2019;19(10):568–586. 31462760 10.1038/s41568-019-0183-z PMC7002027

[87] Kharitonenkov A , Chen Z , Sures I , Wang H , Schilling J , Ullrich A . A family of proteins that inhibit signalling through tyrosine kinase receptors. Nature. 1997;386(6621):181–186. 9062191 10.1038/386181a0

[88] Barclay AN , Brown MH . The SIRP family of receptors and immune regulation. Nat Rev Immunol. 2006;6(6):457–464. 16691243 10.1038/nri1859

[89] Kortlever RM , Sodir NM , Wilson CH , Burkhart DL , Pellegrinet L , Brown Swigart L , et al. Myc cooperates with Ras by programming inflammation and immune suppression. Cell. 2017;171(6):1301–1315.e14. 29195074 10.1016/j.cell.2017.11.013 PMC5720393

[90] Li J , Dong T , Wu Z , Zhu D , Gu H . The effects of MYC on tumor immunity and immunotherapy. Cell Death Dis. 2023;9(1):103. 10.1038/s41420-023-01403-3 PMC10039951 36966168

[91] Wu SY , Xiao Y , Wei JL , Xu XE , Jin X , Hu X , et al. MYC suppresses STING‐dependent innate immunity by transcriptionally upregulating DNMT1 in triple‐negative breast cancer. J Immunother Cancer. 2021;9(7):e002528. 34321275 10.1136/jitc-2021-002528 PMC8320259

[92] Dhanasekaran R , Baylot V , Kim M , Kuruvilla S , Bellovin DI , Adeniji N , et al. MYC and Twist1 cooperate to drive metastasis by eliciting crosstalk between cancer and innate immunity. eLife. 2020;9:9. 10.7554/eLife.50731 PMC6959993 31933479

[93] Sodir NM , Pellegrinet L , Kortlever RM , Campos T , Kwon YW , Kim S , et al. Reversible MYC hypomorphism identifies a key MYC‐dependency in early cancer evolution. Nat Commun. 2022;13(1):6782. 36351945 10.1038/s41467-022-34079-x PMC9646778

[94] Zhang T , Ren Y , Yang P , Wang J , Zhou H . Cancer‐associated fibroblasts in pancreatic ductal adenocarcinoma. Cell Death Dis. 2022;13(10):897. 36284087 10.1038/s41419-022-05351-1 PMC9596464

[95] Karamitopoulou E . Tumour microenvironment of pancreatic cancer: immune landscape is dictated by molecular and histopathological features. Br J Cancer. 2019;121(1):5–14. 31110329 10.1038/s41416-019-0479-5 PMC6738327

[96] Pearce OMT , Delaine‐Smith RM , Maniati E , Nichols S , Wang J , Böhm S , et al. Deconstruction of a metastatic tumor microenvironment reveals a common matrix response in human cancers. Cancer Discov. 2018;8(3):304–319. 29196464 10.1158/2159-8290.CD-17-0284 PMC5837004

[97] Kosan C , Saba I , Godmann M , Herold S , Herkert B , Eilers M , et al. Transcription factor miz‐1 is required to regulate interleukin‐7 receptor signaling at early commitment stages of B cell differentiation. Immunity. 2010;33(6):917–928. 21167753 10.1016/j.immuni.2010.11.028

[98] Staller P , Peukert K , Kiermaier A , Seoane J , Lukas J , Karsunky H , et al. Repression of p15INK4b expression by Myc through association with Miz‐1. Nat Cell Biol. 2001;3(4):392–399. 11283613 10.1038/35070076

[99] van Riggelen J , Müller J , Otto T , Beuger V , Yetil A , Choi PS , et al. The interaction between Myc and Miz1 is required to antagonize TGFbeta‐dependent autocrine signaling during lymphoma formation and maintenance. Genes Dev. 2010;24(12):1281–1294. 20551174 10.1101/gad.585710 PMC2885663

[100] Tesi A , de Pretis S , Furlan M , Filipuzzi M , Morelli MJ , Andronache A , et al. An early Myc‐dependent transcriptional program orchestrates cell growth during B‐cell activation. EMBO Rep. 2019;20(9):e47987. 31334602 10.15252/embr.201947987 PMC6726900

[101] de Pretis S , Kress TR , Morelli MJ , Sabò A , Locarno C , Verrecchia A , et al. Integrative analysis of RNA polymerase II and transcriptional dynamics upon MYC activation. Genome Res. 2017;27(10):1658–1664. 28904013 10.1101/gr.226035.117 PMC5630029

[102] Hsieh AL , Walton ZE , Altman BJ , Stine ZE , Dang CV . MYC and metabolism on the path to cancer. Semin Cell Dev Biol. 2015;43:11–21. 26277543 10.1016/j.semcdb.2015.08.003 PMC4818970

[103] Cairns RA , Harris IS , Mak TW . Regulation of cancer cell metabolism. Nat Rev Cancer. 2011;11(2):85–95. 21258394 10.1038/nrc2981

[104] Stine ZE , Walton ZE , Altman BJ , Hsieh AL , Dang CV . MYC, metabolism, and cancer. Cancer Discov. 2015;5(10):1024–1039. 26382145 10.1158/2159-8290.CD-15-0507 PMC4592441

[105] Le A , Lane AN , Hamaker M , Bose S , Gouw A , Barbi J , et al. Glucose‐independent glutamine metabolism via TCA cycling for proliferation and survival in B cells. Cell Metab. 2012;15(1):110–121. 22225880 10.1016/j.cmet.2011.12.009 PMC3345194

[106] Wise DR , DeBerardinis RJ , Mancuso A , Sayed N , Zhang XY , Pfeiffer HK , et al. Myc regulates a transcriptional program that stimulates mitochondrial glutaminolysis and leads to glutamine addiction. Proc Natl Acad Sci USA. 2008;105(48):18782–18787. 19033189 10.1073/pnas.0810199105 PMC2596212

[107] DePeaux K , Delgoffe GM . Metabolic barriers to cancer immunotherapy. Nat Rev Immunol. 2021;21(12):785–797. 33927375 10.1038/s41577-021-00541-y PMC8553800

[108] Chang CH , Qiu J , O'Sullivan D , Buck MD , Noguchi T , Curtis JD , et al. Metabolic competition in the tumor microenvironment is a driver of cancer progression. Cell. 2015;162(6):1229–1241. 26321679 10.1016/j.cell.2015.08.016 PMC4864363

[109] O'Connell BC , Cheung AF , Simkevich CP , Tam W , Ren X , Mateyak MK , et al. A large scale genetic analysis of c‐Myc‐regulated gene expression patterns. J Biol Chem. 2003;278(14):12563–12573. 12529326 10.1074/jbc.M210462200

[110] Menssen A , Hermeking H . Characterization of the c‐MYC‐regulated transcriptome by SAGE: identification and analysis of c‐MYC target genes. Proc Natl Acad Sci USA. 2002;99(9):6274–6279. 11983916 10.1073/pnas.082005599 PMC122939

[111] Osthus RC , Shim H , Kim S , Li Q , Reddy R , Mukherjee M , et al. Deregulation of glucose transporter 1 and glycolytic gene expression by c‐Myc. J Biol Chem. 2000;275(29):21797–21800. 10823814 10.1074/jbc.C000023200

[112] Feng YB , Xiong Y , Qiao T , Li X , Jia L , Han Y . Lactate dehydrogenase a: a key player in carcinogenesis and potential target in cancer therapy. Cancer Med. 2018;7(12):6124–6136. 30403008 10.1002/cam4.1820 PMC6308051

[113] Shim H , Dolde C , Lewis BC , Wu CS , Dang G , Jungmann RA , et al. c‐Myc transactivation of LDH‐A: implications for tumor metabolism and growth. Proc Natl Acad Sci USA. 1997;94(13):6658–6663. 9192621 10.1073/pnas.94.13.6658 PMC21214

[114] Parks SK , Mueller‐Klieser W , Pouyssegur J . Lactate and acidity in the cancer microenvironment. Annual Review of Cancer Biology. 2020;4(4):141–158.

[115] Sangsuwan R , Thuamsang B , Pacifici N , Allen R , Han H , Miakicheva S , et al. Lactate exposure promotes immunosuppressive phenotypes in innate immune cells. Cell Mol Bioeng. 2020;13(5):541–557. 33184582 10.1007/s12195-020-00652-x PMC7596145

[116] Dietl K , Renner K , Dettmer K , Timischl B , Eberhart K , Dorn C , et al. Lactic acid and acidification inhibit TNF secretion and glycolysis of human monocytes. J Immunol. 2010;184(3):1200–1209. 20026743 10.4049/jimmunol.0902584

[117] Ye HL , Zhou Q , Zheng S . Tumor‐associated macrophages proote progression and the Warburg effect via CCL18/NF‐kB/VCAM‐1 pathway in pancreatic ductal adenocarcinoma. Cell Death Dis. 2018;9:9. 29670110 10.1038/s41419-018-0486-0 PMC5906621

[118] Fischer K , Hoffmann P , Voelkl S , Meidenbauer N , Ammer J , Edinger M , et al. Inhibitory effect of tumor cell‐derived lactic acid on human T cells. Blood. 2007;109(9):3812–3819. 17255361 10.1182/blood-2006-07-035972

[119] Kumagai S , Koyama S , Itahashi K . Lactic acid promotes PD‐1 expression in regulatory T cells in highly glycolytic tumor microenvironments. Cancer Cell. 2022;40(2):201. 35090594 10.1016/j.ccell.2022.01.001

[120] Hensley CT , Wasti AT , DeBerardinis RJ . Glutamine and cancer: cell biology, physiology, and clinical opportunities. J Clin Invest. 2013;123(9):3678–3684. 23999442 10.1172/JCI69600 PMC3754270

[121] Gao P , Tchernyshyov I , Chang TC , Lee YS , Kita K , Ochi T , et al. c‐Myc suppression of miR‐23a/b enhances mitochondrial glutaminase expression and glutamine metabolism. Nature. 2009;458(7239):762–765. 19219026 10.1038/nature07823 PMC2729443

[122] Leone RD , Zhao L , Englert JM , Sun IM , Oh MH , Sun IH , et al. Glutamine blockade induces divergent metabolic programs to overcome tumor immune evasion. Science. 2019;366(6468):1013–1021. 31699883 10.1126/science.aav2588 PMC7023461

[123] Guo C , You Z , Shi H , Sun Y , du X , Palacios G , et al. SLC38A2 and glutamine signalling in cDC1s dictate anti‐tumour immunity. Nature. 2023;620(7972):200–208. 37407815 10.1038/s41586-023-06299-8 PMC10396969

[124] Kumar A , Yarosz EL , Andren A , Zhang L , Lyssiotis CA , Chang CH . NKT cells adopt a glutamine‐addicted phenotype to regulate their homeostasis and function. Cell Rep. 2022;41(4):111516. 36288696 10.1016/j.celrep.2022.111516 PMC9664378

[125] Watson MJ , Vignali PDA , Mullett SJ , Overacre‐Delgoffe AE , Peralta RM , Grebinoski S , et al. Metabolic support of tumour‐infiltrating regulatory T cells by lactic acid. Nature. 2021;591(7851):645. 33589820 10.1038/s41586-020-03045-2 PMC7990682

[126] Nakaya M , Xiao Y , Zhou X , Chang JH , Chang M , Cheng X , et al. Inflammatory T cell responses rely on amino acid transporter ASCT2 facilitation of glutamine uptake and mTORC1 kinase activation. Immunity. 2014;40(5):692–705. 24792914 10.1016/j.immuni.2014.04.007 PMC4074507

[127] Klysz D , Tai X , Robert PA , Craveiro M , Cretenet G , Oburoglu L , et al. Glutamine‐dependent alpha‐ketoglutarate production regulates the balance between T helper 1 cell and regulatory T cell generation. Sci Signal. 2015;8(396):ra97. 26420908 10.1126/scisignal.aab2610

[128] Nguyen HV , Vandenberg CJ , Ng AP , Robati MR , Anstee NS , Rimes J , et al. Development and survival of MYC‐driven lymphomas require the MYC antagonist MNT to curb MYC‐induced apoptosis. Blood. 2020;135(13):1019–1031. 31978211 10.1182/blood.2019003014 PMC7118401

[129] Wilde BR , Ye Z , Lim TY , Ayer DE . Cellular acidosis triggers human MondoA transcriptional activity by driving mitochondrial ATP production. eLife. 2019;8:8. 10.7554/eLife.40199 PMC6363388 30717828

[130] Carroll PA , Freie BW , Cheng PF , Kasinathan S , Gu H , Hedrich T , et al. The glucose‐sensing transcription factor MLX balances metabolism and stress to suppress apoptosis and maintain spermatogenesis. PLoS Biol. 2021;19(10):e3001085. 34669700 10.1371/journal.pbio.3001085 PMC8528285

[131] Carroll PA , Diolaiti D , McFerrin L , Gu H , Djukovic D , du J , et al. Deregulated Myc requires MondoA/mlx for metabolic reprogramming and tumorigenesis. Cancer Cell. 2015;27(2):271–285. 25640402 10.1016/j.ccell.2014.11.024 PMC4326605

[132] Patange S , Ball DA , Wan Y , Karpova TS , Girvan M , Levens D , et al. MYC amplifies gene expression through global changes in transcription factor dynamics. Cell Rep. 2022;38(4):110292. 35081348 10.1016/j.celrep.2021.110292 PMC8849550

[133] Nie Z , Hu G , Wei G , Cui K , Yamane A , Resch W , et al. c‐Myc is a universal amplifier of expressed genes in lymphocytes and embryonic stem cells. Cell. 2012;151(1):68–79. 23021216 10.1016/j.cell.2012.08.033 PMC3471363

[134] Crossley MP , Song C , Bocek MJ , Choi JH , Kousouros JN , Sathirachinda A , et al. R‐loop‐derived cytoplasmic RNA‐DNA hybrids activate an immune response. Nature. 2023;613(7942):187–194. 36544021 10.1038/s41586-022-05545-9 PMC9949885

[135] Weinreb JT , Ghazale N , Pradhan K , Gupta V , Potts KS , Tricomi B , et al. Excessive R‐loops trigger an inflammatory cascade leading to increased HSPC production. Dev Cell. 2021;56(5):627–640.e5. 33651979 10.1016/j.devcel.2021.02.006 PMC8258699

[136] Ahmad S , Mu X , Yang F , Greenwald E , Park JW , Jacob E , et al. Breaching self‐tolerance to Alu duplex RNA underlies MDA5‐mediated inflammation. Cell. 2018;172(4):797–810.e13. 29395326 10.1016/j.cell.2017.12.016 PMC5807104

[137] Morel KL , Sheahan AV , Burkhart DL , Baca SC , Boufaied N , Liu Y , et al. EZH2 inhibition activates a dsRNA–STING–interferon stress axis that potentiates response to PD‐1 checkpoint blockade in prostate cancer. Nat Can. 2021;2(4):444–456. 10.1038/s43018-021-00185-w PMC8061902 33899001

[138] Ishizuka JJ , Manguso RT , Cheruiyot CK , Bi K , Panda A , Iracheta‐Vellve A , et al. Loss of ADAR1 in tumours overcomes resistance to immune checkpoint blockade. Nature. 2019;565(7737):43–48. 30559380 10.1038/s41586-018-0768-9 PMC7241251

[139] Topper MJ , Vaz M , Chiappinelli KB , DeStefano Shields CE , Niknafs N , Yen RWC , et al. Epigenetic therapy ties MYC depletion to reversing immune evasion and treating lung cancer. Cell. 2017;171(6):1284–1300.e21. 29195073 10.1016/j.cell.2017.10.022 PMC5808406

[140] Herold S , Hock A , Herkert B , Berns K , Mullenders J , Beijersbergen R , et al. Miz1 and HectH9 regulate the stability of the checkpoint protein, TopBP1. EMBO J. 2008;27(21):2851–2861. 18923429 10.1038/emboj.2008.200 PMC2580782

[141] Urbanski L , Brugiolo M , Park SH , Angarola BL , Leclair NK , Yurieva M , et al. MYC regulates a pan‐cancer network of co‐expressed oncogenic splicing factors. Cell Rep. 2022;41(8):111704. 36417849 10.1016/j.celrep.2022.111704 PMC9731204

[142] Koh CM , Bezzi M , Low DHP , Ang WX , Teo SX , Gay FPH , et al. MYC regulates the core pre‐mRNA splicing machinery as an essential step in lymphomagenesis. Nature. 2015;523(7558):96–100. 25970242 10.1038/nature14351

[143] Liu L , Ulbrich J , Müller J , Wüstefeld T , Aeberhard L , Kress TR , et al. Deregulated MYC expression induces dependence upon AMPK‐related kinase 5. Nature. 2012;483(7391):608–612. 22460906 10.1038/nature10927

[144] Iwai K , Yaguchi M , Nishimura K , Yamamoto Y , Tamura T , Nakata D , et al. Anti‐tumor efficacy of a novel CLK inhibitor via targeting RNA splicing and MYC‐dependent vulnerability. EMBO Mol Med. 2018;10(6):e8289. 29769258 10.15252/emmm.201708289 PMC5991599

[145] Lu SX , de Neef E , Thomas JD , Sabio E , Rousseau B , Gigoux M , et al. Pharmacologic modulation of RNA splicing enhances anti‐tumor immunity. Cell. 2021;184(15):4032–4047.e31. 34171309 10.1016/j.cell.2021.05.038 PMC8684350

[146] Reisländer T , Lombardi EP , Groelly FJ , Miar A , Porru M , di Vito S , et al. BRCA2 abrogation triggers innate immune responses potentiated by treatment with PARP inhibitors. Nat Commun. 2019;10(1):3143. 31316060 10.1038/s41467-019-11048-5 PMC6637138

[147] Wolf C , Rapp A , Berndt N , Staroske W , Schuster M , Dobrick‐Mattheuer M , et al. RPA and Rad51 constitute a cell intrinsic mechanism to protect the cytosol from self DNA. Nat Commun. 2016;7:7. 10.1038/ncomms11752 PMC4895045 27230542

[148] Mackenzie KJ , Carroll P , Martin CA , Murina O , Fluteau A , Simpson DJ , et al. cGAS surveillance of micronuclei links genome instability to innate immunity. Nature. 2017;548(7668):461–465. 28738408 10.1038/nature23449 PMC5870830

[149] Wang C , Zhang J , Yin J , Gan Y , Xu S , Gu Y , et al. Alternative approaches to target Myc for cancer treatment. Signal Transduct Target Ther. 2021;6(1):117. 33692331 10.1038/s41392-021-00500-y PMC7946937

[150] Ciesla M , Ngoc PCT , Cordero E , Martinez ÁS , Morsing M , Muthukumar S , et al. Oncogenic translation directs spliceosome dynamics revealing an integral role for SF3A3 in breast cancer. Mol Cell. 2021;81(7):1453–1468 e12. 33662273 10.1016/j.molcel.2021.01.034

[151] Hsu TYT , Simon LM , Neill NJ , Marcotte R , Sayad A , Bland CS , et al. The spliceosome is a therapeutic vulnerability in MYC‐driven cancer. Nature. 2015;525(7569):384. 26331541 10.1038/nature14985 PMC4831063

[152] Han H , Jain AD , Truica MI , Izquierdo‐Ferrer J , Anker JF , Lysy B , et al. Small‐molecule MYC inhibitors suppress tumor growth and enhance immunotherapy. Cancer Cell. 2019;36(5):483–497.e15. 31679823 10.1016/j.ccell.2019.10.001 PMC6939458

[153] Truica MI , Burns MC , Han H , Abdulkadir SA . Turning up the heat on MYC: Progress in small‐molecule inhibitors. Cancer Res. 2021;81(2):248–253. 33087323 10.1158/0008-5472.CAN-20-2959 PMC7855142

[154] Wang H , Hammoudeh DI , Follis AV , Reese BE , Lazo JS , Metallo SJ , et al. Improved low molecular weight Myc‐max inhibitors. Mol Cancer Ther. 2007;6(9):2399–2408. 17876039 10.1158/1535-7163.MCT-07-0005

[155] Yang C , Liu Y , Hu Y , Fang L , Huang Z , Cui H , et al. Myc inhibition tips the immune balance to promote antitumor immunity. Cell Mol Immunol. 2022;19(9):1030–1041. 35962189 10.1038/s41423-022-00898-7 PMC9424194

[156] Lin F‐T , Liu K , Garan LAW , Folly‐Kossi H , Song Y , Lin SJ , et al. A small‐molecule inhibitor of TopBP1 exerts anti‐MYC activity and synergy with PARP inhibitors. Proc Natl Acad Sci. 2023;120(44):e2307793120. 37878724 10.1073/pnas.2307793120 PMC10622895

[157] Ai Y , Hwang L , MacKerell AD Jr , Melnick A , Xue F . Progress toward B‐cell lymphoma 6 BTB domain inhibitors for the treatment of diffuse large B‐cell lymphoma and beyond. J Med Chem. 2021;64(8):4333–4358. 33844535 10.1021/acs.jmedchem.0c01686 PMC8168288

[158] Wu X , Nelson M , Basu M , Srinivasan P , Lazarski C , Zhang P , et al. MYC oncogene is associated with suppression of tumor immunity and targeting Myc induces tumor cell immunogenicity for therapeutic whole cell vaccination. J Immunother Cancer. 2021;9(3):e001388. 33757986 10.1136/jitc-2020-001388 PMC7993333

[159] Soucek L , Whitfield JR , Sodir NM , Massó‐Vallés D , Serrano E , Karnezis AN , et al. Inhibition of Myc family proteins eradicates KRas‐driven lung cancer in mice. Genes Dev. 2013;27(5):504–513. 23475959 10.1101/gad.205542.112 PMC3605464

[160] Garralda E , Beaulieu ME , Moreno V , Casacuberta‐Serra S , Martínez‐Martín S , Foradada L , et al. MYC targeting by OMO‐103 in solid tumors: a phase 1 trial. Nat Med. 2024;30(3):762–771. 38321218 10.1038/s41591-024-02805-1 PMC10957469

[161] Jung LA , Gebhardt A , Koelmel W , Ade CP , Walz S , Kuper J , et al. OmoMYC blunts promoter invasion by oncogenic MYC to inhibit gene expression characteristic of MYC‐dependent tumors. Oncogene. 2017;36(14):1911–1924. 27748763 10.1038/onc.2016.354

[162] Casacuberta Serra S , Martínez‐Martín S , González‐Larreategui Í , López‐Estévez S , Jauset T , Zacarías‐Fluck M , et al. 129 (PB119) – MYC inhibition by OMO‐103 induces immune cell recruitment in preclinical models of NSCLC and modulates the cytokine and chemokine profiles of phase I patients showing stable disease. Eur J Cancer. 2022;174:S46–S47.

[163] Zhang P , Wu X , Basu M , Dong C , Zheng P , Liu Y , et al. MYCN amplification is associated with repressed cellular immunity in Neuroblastoma: an in Silico immunological analysis of TARGET database. Front Immunol. 2017;8:1473. 29163537 10.3389/fimmu.2017.01473 PMC5675839

[164] Sullivan DK , Deutzmann A , Yarbrough J , Krishnan MS , Gouw AM , Bellovin DI , et al. MYC oncogene elicits tumorigenesis associated with embryonic, ribosomal biogenesis, and tissue‐lineage dedifferentiation gene expression changes. Oncogene. 2022;41(45):4960–4970. 36207533 10.1038/s41388-022-02458-9 PMC10257951

